# Comparative *In Vitro* Toxicity Profile of Electronic and Tobacco Cigarettes, Smokeless Tobacco and Nicotine Replacement Therapy Products: E-Liquids, Extracts and Collected Aerosols

**DOI:** 10.3390/ijerph111111325

**Published:** 2014-10-30

**Authors:** Manoj Misra, Robert D. Leverette, Bethany T. Cooper, Melanee B. Bennett, Steven E. Brown

**Affiliations:** Lorillard Tobacco Company, A.W. Spears Research Center, 420 North English Street, Greensboro, North Carolina 27405, USA; E-Mails: rleverette@lortobco.com (R.D.L.); bethanycooper@lortobco.com (B.T.C.); mbennett@lortobco.com (M.B.B.); sbrown@lortobco.com (S.E.B.)

**Keywords:** e-cigarette, snus, snuff, e-liquid, aerosol, cytotoxicity, mutagenicity, inflammation, condensate, *in vitro*

## Abstract

The use of electronic cigarettes (e-cigs) continues to increase worldwide in parallel with accumulating information on their potential toxicity and safety. In this study, an *in vitro* battery of established assays was used to examine the cytotoxicity, mutagenicity, genotoxicity and inflammatory responses of certain commercial e-cigs and compared to tobacco burning cigarettes, smokeless tobacco (SLT) products and a nicotine replacement therapy (NRT) product. The toxicity evaluation was performed on e-liquids and pad-collected aerosols of e-cigs, pad-collected smoke condensates of tobacco cigarettes and extracts of SLT and NRT products. In all assays, exposures with e-cig liquids and collected aerosols, at the doses tested, showed no significant activity when compared to tobacco burning cigarettes. Results for the e-cigs, with and without nicotine in two evaluated flavor variants, were very similar in all assays, indicating that the presence of nicotine and flavors, at the levels tested, did not induce any cytotoxic, genotoxic or inflammatory effects. The present findings indicate that neither the e-cig liquids and collected aerosols, nor the extracts of the SLT and NRT products produce any meaningful toxic effects in four widely-applied *in vitro* test systems, in which the conventional cigarette smoke preparations, at comparable exposures, are markedly cytotoxic and genotoxic.

## 1. Introduction

The typical commercial electronic cigarette (e-cig) is comprised of three major components: a rechargeable or disposable battery, a heating element that generates an inhalable aerosol, and an associated switch or puff-activated circuitry. The circuitry serves to produce the aerosol only during the active puffing cycle, essentially eliminating sidestream emissions from the device during usage. The typical commercial e-cig also contains a liquid solution containing aerosol-forming excipients such as glycerol and/or propylene glycol, flavoring materials and, optionally, nicotine. This solution is usually delivered from a small reservoir by capillary wicking to the heating zone to affect the generation of an aerosol that superficially resembles cigarette smoke in appearance. A great variety of e-cig sizes, configurations, liquid formulations and designs are emerging on a continual basis in this rapidly-developing worldwide marketplace in response to users’ evolving personal preferences.

As such, the popularity and sales volume of e-cigs continue to increase worldwide [1,2], and there is a need for a fuller scientific understanding of the potential benefits or risks that e-cigs may have, both to individual users as well as the general smoking and nonsmoking populations. A contemporary framework for assessing the relative risks and benefits to both individuals and populations must necessarily include the characterization of any potential toxicological hazard inherent to a product, and a consideration of those properties against those of other available alternative products. The individual risks to smokers and the harm to populations resulting from conventional cigarette smoking are very well understood and extensively documented [3]; however, the use of alternative products, such as electronic cigarettes, holds potential as an effective approach to advancing the public health amongst adult smokers in the near term [4,5,6].

It is well understood that tobacco cigarettes produce a multitude of harmful and toxic constituents that together induce deleterious health effects including chronic obstructive pulmonary disease (COPD), cardiovascular disease (CVD) and cancer [3]. Conversely, e-cigs do not burn tobacco and do not deliver harmful constituents in the numbers or in nearly the quantities that are found in the smoke of conventional tobacco cigarettes [7]. A recent review reporting on chemical, toxicological (mostly cytotoxicity studies on established cell lines) and clinical studies clearly indicates that e-cig liquids and aerosols contain far less and fewer chemicals, induce significantly less cytotoxicity or adverse effects, and result in considerably reduced cardiovascular and respiratory functional effects than are reported for tobacco cigarettes [8]. In prior investigations of aqueous extract of e-cig aerosols in mammalian fibroblast cells [9] and myocardial cells [10], some moderate cytotoxicity was observed for certain flavoring compounds found in the tested products. However, all such studies, to date, have reported the tested e-cig liquids and aerosols to be markedly less toxic than extracts prepared from the smoke of conventional cigarettes.

This study utilized an *in vitro* battery of established assays to examine the cytotoxicity (Neutral Red Uptake; NRU), mutagenicity (reverse bacterial mutagenicity test; Ames), genotoxicity (micronucleus formation; MN) and inflammatory effect (cytokine IL-8 release: IL-8) in cells exposed to preparations of various tobacco cigarettes, smokeless tobacco (SLT), nicotine replacement therapy (NRT) products, and commercial electronic cigarettes. Pre-incubation and in-media exposure methods were adopted for e-liquids, aqueous extracts of SLT, NRT products, and pad-collected aerosols from e-cigs as well as pad-collected smoke from tobacco cigarettes.

To date, no systematic toxicity studies have been reported that directly compared e-cig with SLT, NRT, and tobacco cigarettes. Therefore, the present comprehensive multi-endpoint study of a variety of tobacco and nicotine delivery products that have been previously assigned different positions on a risk continuum [11,12] was designed to address the following:

1. Toxicity of e-cig liquids;
2. Toxicity of SLT products;
3. Toxicity of a NRT lozenge product;
4. Toxicity of pad-collected particulate matter from freshly-generated smoke and aerosols from tobacco cigarettes and e-cigs, respectively.

## 2. Materials and Methods

### 2.1. Chemicals and Methods

All chemicals were purchased from Sigma-Aldrich (St. Louis, MO, USA) unless otherwise stated. Recommendations from Cooperation Centre for Scientific Research Relative to Tobacco (CORESTA) were followed for the selection of toxicological assays used in this study [13].

### 2.2. Product Characterization

Commercial blu e-cigs containing glycerol-based e-liquids, with and without nicotine and two market leader flavors (Classic Tobacco and Magnificent Menthol), were used in this study. For comparative purposes, tobacco burning cigarettes (Kentucky Reference 3R4F, 1R5F and Marlboro Gold), SLT products (Marlboro Snus, Copenhagen Snuff) and a NRT product (Nicorette Lozenge) were also tested. The products used in the study, their general specifications and the level of nicotine measured in test samples are detailed in Table 1.

### 2.3. E-Liquid Extraction

The e-liquids were extracted from the wicking material located inside the cartomizer for both rechargeable and disposable e-cigs under aseptic conditions. The mouth-end plug of e-cigs was removed and the polyester wicking material was removed with sterilized stainless steel forceps. The wet wicking material was then placed in a sterile 20 mL plastic syringe tipped with a sterile 0.45 µm pore size syringe filter. The e-liquids from the wet wicking material were extracted by pushing the syringe plunger and collected in a sterilized test tube. About 1.0 mL of e-liquid was extracted from each e-cig. Subsequently, the e-liquids were diluted and delivered to the respective test systems.

**Table 1:** Product Characterization.

Product Class	Name/Description	Abbreviation	Lot #	Product Label Nicotine (mg)	Nicotine Measured in Samples (mg/mL)
Tobacco Cigarettes	Kentucky Reference Cigarettes	3R4F	--	0.8	2.08 *
		1R5F	--	0.2	1.27 ± 0.10
	Marlboro Gold, 72 mm	Marlboro Gold	V128Z33B4	0.68	1.96 ± 0.08
Electronic Cigarettes (e-cig)	Control e-cig	N/A	--	0	0 ± 0
	blu™ e-cigs Classic Tobacco No Nicotine (Rechargeable)	blu CT-Ø	0248	0	0 ± 0
	blu™ e-cigs Classic Tobacco High Nicotine (Cigalike)	blu CT-High	270/404	24.0	17.93 ± 0.34
	blu™ e-cigs Magnificent Menthol No Nicotine (Rechargeable)	blu MM-Ø	237	0	0 ± 0
	blu™ e-cigs Magnificent Menthol High Nicotine (Cigalike)	blu MM-High	404	24.0	20.43 ± 0.41
Smokeless Tobacco (SLT)	Marlboro^®^ Snus	N/A	N335X0X50	15.7	0.42 ± 0.14
	Copenhagen^®^ Snuff	N/A	NEI31755H	10.6	0.46 ± 0.03
Nicotine Replacement Therapy (NRT)	Nicorette^®^ Lozenge	N/A	13780	4.0	0.10 ± 0.03

* Only one sample value.

### 2.4. Pad-Collected Aerosols for Tobacco Cigarettes and E-Cigs

All tobacco cigarettes were conditioned at 60% relative humidity at 24°C for at least 18 h prior to machine smoking. E-Cig batteries were charged immediately prior to use (rechargeable only). The e-liquid from the control e-cig contained a glycerol/water mixture, without flavors or nicotine, similar to the tested commercial products.

It has been suggested that realistic tobacco cigarette smoking, as well as the e-cig vaping profile, are more intense than the ISO machine smoking profile (35 mL puff volume, 2 s draw, 60 s puff interval). In the absence of a standardized vaping profile for e-cigs and our intention to compare e-cig toxicity with conventional cigarette toxicity, this study employed the Canadian Intense (CI) puffing conditions. Both tobacco and e-cigs were smoked on a VITROCELL^®^ VC10 smoking robot (VITROCELL Systems, Waldkirch, Germany) under the CI puffing conditions: 55 mL puff volume, 2 s draw, 30 s puff interval, and 100% blocked air dilution in the case of tobacco cigarettes [14]. Wet Total Particulate Matter (WTPM) and e-cig aerosols were collected on Cambridge glass fiber filter pads, which capture in excess of 99% of cigarette smoke particulate matter. The filters were extracted into either dimethylsulfoxide (DMSO) for tobacco smoke or phosphate buffered saline (PBS) for e-cig aerosols, both to a final concentration of 40 mg/mL (*w/v*) and stored at −80 °C prior to analysis.

### 2.5. Aqueous Extract of Smokeless Tobacco *(*SLT*)* and Nicotine Replacement Therapy *(*NRT*)* Products

Aqueous extracts from commercially available products obtained at retail outlets were prepared based on previously reported methods [15]. Products were suspended in PBS at 80 mg/mL (Dulbecco’s PBS, #14040, +MgCl_2_ +CaCl_2_, Gibco, Grand Island, NY, USA). The suspension was incubated at 37 °C for 21–24 h, shaking at 150 rpm on a shaker incubator. The final suspension was then centrifuged at 12,000 g for 10 min to remove particulates, filter sterilized, aliquoted and stored at −80 °C prior to analysis.

### 2.6. Nicotine Measurement

The level of nicotine in e-liquids and pad-collected smoke and aerosols was quantified using Gas Chromatography-Flame Ionization Detection (GC-FID) instrumentation with a six point calibration utilizing a nicotine standard concentration range [16]. The method precision “variability” was 0.3%–0.7%, method accuracy was 97.4%–98.6%, method LOD was 0.0524 mg/g and method LOQ was 0.1040 mg/g.

### 2.7. Cell Culture

Human lung epithelial carcinoma cells A549 (ATCC# CCL-185) were plated in 96-well plates in 200 μL per well of complete medium (Ham’s F-12K medium with 10% heat-inactivated fetal bovine serum (FBS), 2 mM L-glutamine and 0.01 mg/mL gentamicin) at a seeding density of 75,000 cells/mL and allowed to attach and grow overnight at 37 °C in an atmosphere of 5% CO_2_ prior to exposures.

Chinese hamster ovary cells CHO-K1 (ATCC# CCL-61) were seeded in 96-well plates at 2500 cells/well in complete growth medium (Ham’s F-12K medium with 10% FBS and 0.01 mg/mL gentamicin) and allowed to attach and grow overnight (37 °C, 5% CO_2_) prior to exposures.

### 2.8. Cell Treatment

Cells were treated for approximately 24 h with increasing levels of e-liquids, aqueous extracts, WTPM or pad-collected e-cig aerosols in fresh complete cell media prior to any toxicological evaluations. The cellular treatment dose range used for e-cigs (e-liquids and pad-collected aerosols) was 0–20 mg/mL and for tobacco cigarettes 0–0.5 mg/mL. The doses utilized for tobacco burning cigarette samples were based on dose range finding experiments that demonstrated high cytotoxicity occurring at or above 0.5 mg/mL. Solubility limitations of e-liquids were observed at doses beyond 20 mg/mL; therefore, doses above 20 mg/mL were not utilized in this study. The cellular treatment dose range used for SLT and NRT samples was 0–27 mg/mL, which incorporated the dose range previously utilized for smokeless tobacco products [15]. The toxicological responses were normalized with their respective vehicle controls, either DMSO for tobacco burning cigarettes or culture medium for all other samples.

### 2.9. Cytotoxicity and IL-8 Assay

Following cellular treatment with samples, a 200 µL aliquot of the exposure medium was taken from each well and processed for IL-8 analysis, and the cells adhered to the wells were processed for the cytotoxicity assay.

Cytotoxicity was measured in A549 cells by the NRU method [17,18]. In brief, the cell treatment medium was replaced with 1.5% (*v/v*) neutral red dye in fresh serum-free complete medium and incubated for 2.5 h. The plates were then washed and the cell-incorporated neutral red dye was released and quantified by measuring absorbance at 540 nm on an Infinite M200 Pro spectrophotometer (TECAN, Morrisville, NC, USA). The EC_50_ for NRU (mg/mL) was calculated and compared using GraphPad Prism v. 5.02 (two tailed; for comparisons, statistical significance @ *p* < 0.05).

The release of cytokine IL-8 was quantified [19] in cellular medium with an ELISA detection kit (Abazyme, Inc., MA, USA) by measuring absorbance at 450 on an Infinite M200 Pro spectrophotometer (TECAN). Results for the IL-8 release are reported as % vehicle control and compared using GraphPad Prism v. 5.02 (two tailed; for comparisons, statistical significance @ *p* < 0.05).

### 2.10. Bacterial Mutagenesis Assay

Ames reverse bacterial mutagenicity assays were conducted with the pre-incubation modification [20,21] in strains TA98 and TA100 with S9 activation. Aroclor-induced Sprague-Dawley rat liver S9 post-mitochondrial supernatant (Moltox, Inc., Boone, NC, USA), in 0.154 M KCl, was used for the S9-cocktail (0.1 M phosphate buffer, pH 7.4; 8 mM MgCl_2_, 33 mM KCl, 5 mM glucose-6-phosphate (G-6-P), 4 mM nicotinamide adenine diphosphate (NADP), 5% (*v/v*) S9-fraction).

The e-cig and SLT/NRT sample dose ranges utilized for the Ames reverse bacterial mutagenicity assay were 0–48 mg/mL and 0–3.2 mg/mL, respectively, due to the sample solubility and sample volume limits of the Ames exposure system. This SLT/NRT dose range is similar to the dose range previously reported for smokeless tobacco product extracts tested in the Ames assay [15]. The control sample for e-cig liquids, e-cig pad-collected aerosols, SLTs and NRT was PBS and the control sample for WTPM from tobacco burning cigarettes was DMSO.

Exposures of *Salmonella* tester strains were performed as follows: 100 μL of an overnight culture, 500 μL of the S9-mix, plus 25 µL sample were combined in a sterile tube, capped and shaken at 250 rpm for 20 min at 37 °C prior to the addition of 2.5 mL of histidine/biotin top agar and plated onto minimal glucose agar plates. Revertant colonies were counted after 48 hours of incubation at 37 °C. All exposures were conducted in triplicate in a minimum of two independent experiments. All colonies were counted with an automated colony counter, Synbiosis ProtoCOL3 (Frederick, MD, USA). Activity reported as revertants per mg was calculated from the linear portion of the dose response curve and compared using GraphPad Prism v. 5.02 (slope analysis, two tailed; for comparisons, statistical significance @ *p* < 0.05).

### 2.11. Micronucleus Assay

The *in vitro* MN assay was performed in CHO-K1cells as previously described [22], utilizing the MN HitKit-K11-0001-1 (Thermo Fisher Scientific, Pittsburgh, PA, USA). Cells were exposed to samples in the absence of S9 for 20 ± 2 h followed by treatment with the cytokinesis blocking agent, cytochalasin B. Cell viability was determined by the cytokinesis-block proliferation index (CBPI). MN frequency (%MN) was determined on a Cellomics^®^ ArrayScan^®^ VTi (Pittsburg, PA, USA) using the Micronucleus Bioapplication, V.4 software. Activity is reported as % Control and compared using GraphPad Prism v. 5.02 (two tailed; for comparisons, statistical significance ^@^ *p* < 0.05).

## 3. Results and Discussion

This *in vitro* comparative toxicological study was designed to evaluate e-liquids extracted from commercial e-cig products and pad-collected aerosols and smoke delivered by laboratory machine smoking of e-cigs and combustible tobacco cigarettes. Although several *in vitro* tests are routinely used and accepted by regulatory authorities, there are inherent limitations which affect the usefulness of the assays to predict toxicity potential of a substance *in vivo*, and especially in humans. Given that no single *in vitro* test can fully replicate *in vivo* test results, a battery of *in vitro* tests with a high concordance to *in vivo* models has the potential to establish a weight-of-evidence approach for evaluating the biological impact associated with e-cigs. In addition, the *in vitro* toxicological analysis of appropriate comparative product types further provides context for results that otherwise may be misleading or lack relevancy to the determination of biological activity.

A battery of toxicological endpoints that have been amply demonstrated to appropriately characterize the responses to cigarette smoke preparations was selected to provide points of reference in terms of the cytotoxicity, mutagenicity, genotoxicity, and inflammatory responses elicited by the tested e-cigs.

Genotoxicity (Ames and MN formation) testing is an important part of the hazard assessment of a chemical for regulatory purposes and has been demonstrated to have very high concordance with rodent carcinogenicity or *in vivo* genotoxicity when tested together [23]. Additionally, inadequate resolution of inflammation and uncontrolled inflammatory reactions can evoke a state of chronic inflammation, which is a common etiologic factor for various human respiratory lesions, including cancer [24,25].

A list of all products evaluated in this study is detailed in Table 1. This study utilized the CI smoking profile to smoke both e-cigs as well as tobacco cigarettes. As a basis for comparative analysis, this study evaluated the toxicological impact of traditional tobacco products, commercial Marlboro Gold and two Kentucky reference cigarettes, four blu e-cigs, Copenhagen Snuff, Marlboro Snus, and Nicorette Lozenge. In addition to comparing products in different classes (tobacco cigarette, e-cig, SLT and NRT), the toxicological impact of nicotine using e-cigs with and without nicotine was also investigated.

### 3.1. E-Liquids, Smokeless Tobaccos *(*SLTs*)* and Nicotine Replacement Therapy *(*NRT*)*: Cytotoxicity

**Figure 1:**
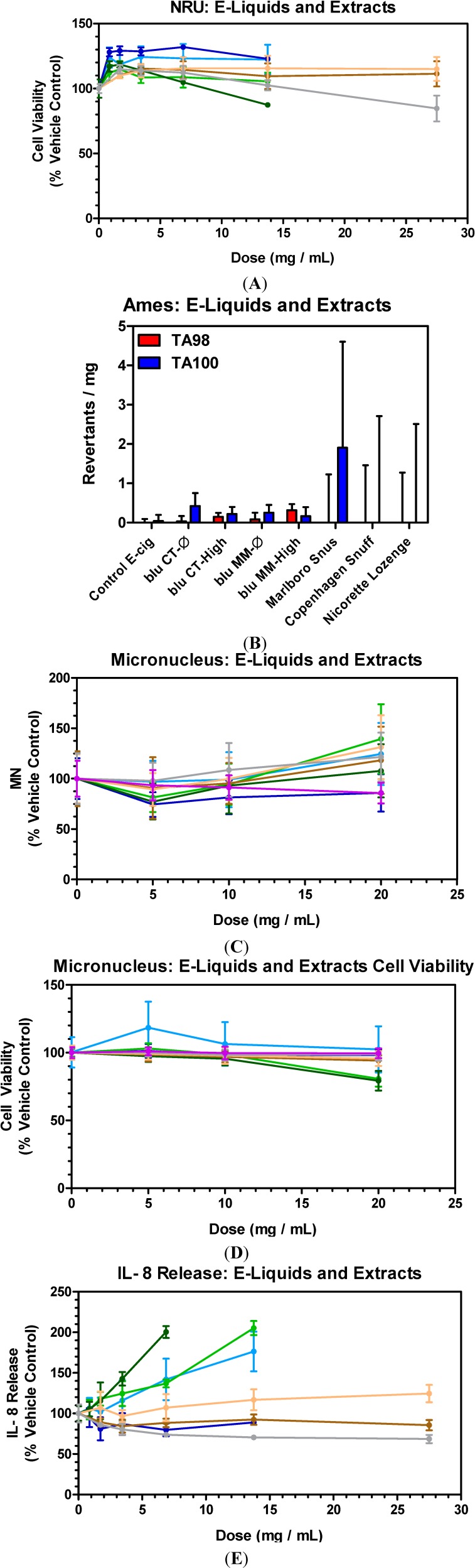
*In vitro* activity of e-cig liquids, smokeless tobacco and lozenge aqueous extracts in NRU (**A**), Ames (**B**), MN (**C** and **D**) and IL-8 (**E**). NRU, MN and IL-8 data reported as % vehicle control, PBS in the case of e-liquids, SLT and NRT aqueous extracts. Data points in each plot represent the mean values ± SD from a minimum of two (2) independent experiments. MN cell viability (**D**) shown to verify lack of MN induction is not due to cytotoxicity at higher doses. (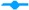) blu CT-Ø; (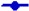) blu CT-High; (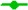) blu MM-Ø; (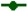) blu MM-High; (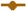) Marlboro Snus; (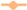) Copenhagen Snuff; (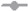) Nicorette Lozenge; (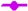) Control e-cig.

The toxicological response of e-liquids and aqueous extracts of SLT and NRT products was evaluated in A549 cells and is shown in Figure 1A–E. No cytotoxicity was observed for any of the e-liquids, as well as for all SLT and NRT products tested up to their respective highest sample doses (Figure 1A).

Similarly, Bahl *et al*. reported little or no cytotoxicity for most of the 35 commercial refill liquids for e-cigs previously tested in human lung fibroblast cells [26]. This study utilized two blu e-cig market leading flavors, classic tobacco and magnificent menthol. To compare the dose levels, the maximum e-liquid dose utilized by Bahl *et al*. [26] was 12.6 mg/mL, equivalent to the highest dose of 1% (*v/v*), assuming a 100 µL MTT assay volume and an e-liquid density of about 1.22 g/mL. Additionally, Bahl *et al*. [26] also reported toxicity to adjacent wells at 10% (*v/v*) e-liquid dilutions, equivalent to approximately 126 mg/mL; however, the present study did not reveal any vapor toxicity from e-liquids at doses as high as 27 mg/mL to adjacent wells. The observed cytotoxicity in adjacent wells [26] may be a result of higher e-liquid concentrations used by Bahl *et al*. or the volatility of specific e-liquid ingredients or flavors released while incubating at 37 ºC, or the possibility of differential susceptibility of the lung fibroblasts used by Bahl *et al.* and the A549 cells used in this study.

**Figure 2:**
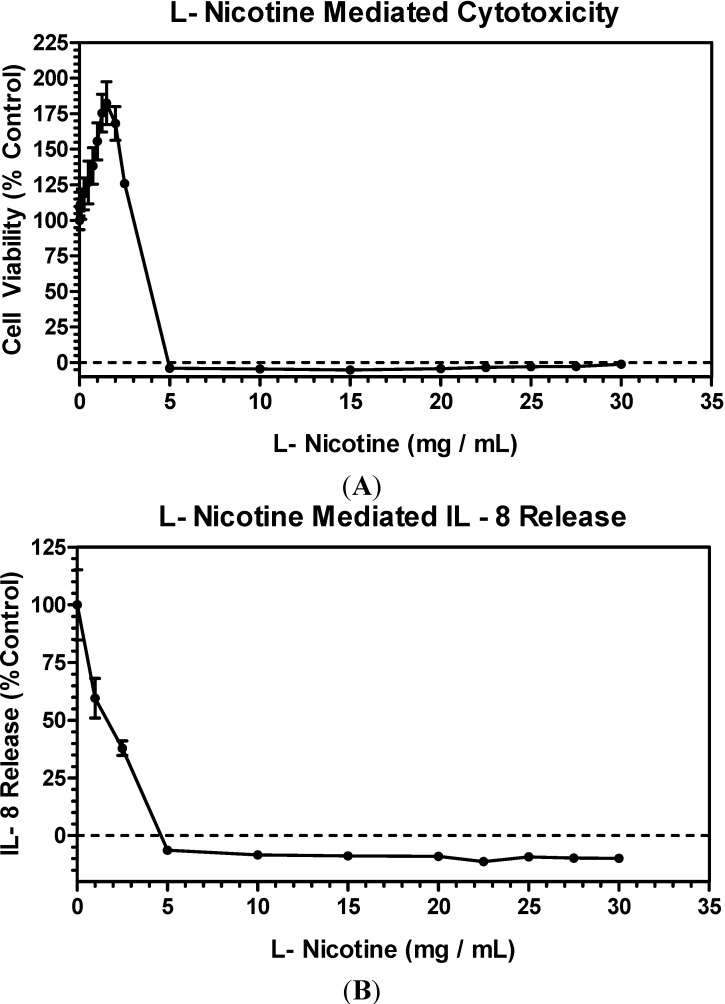
Effects of l-nicotine on cytotoxicity (**A**) NRU and inflammation (**B**) IL-8 in A549 cells. Data points in each plot represent the mean values ± SD from a minimum of two (2) independent experiments.

At the lower doses of e-liquids utilized in this study, an increase in cell viability was observed (Figure 1A) which was also evident with cells treated with lower doses of pure nicotine (Figure 2A). This increase in cellular viability was primarily associated with higher cellular proliferation and cellular protection mediated by the low level of nicotine exposure [27]. Therefore, there may be an association between the lower nicotine present in e-cig liquids and increased cellular viability, thus cellular protection.

### 3.2. E-Liquids, Smokeless Tobaccos *(*SLTs*)* and Nicotine Replacement Therapy *(*NRT*)*: Mutagenicity

The Ames test, also known as the bacterial reverse mutation assay, is widely used for the determination of a compound’s ability to induce mutations and has been shown to have a high predictive value with rodent carcinogenicity tests [28].

The activity of e-liquids, SLT and NRT extracts in the Ames assay is shown in Figure 1B. The control e-cig sample containing water/glycerol and the PBS control sample (SLT and NRT) did not induce any revertants above baseline and were within the variability range of this assay. No significant induction in the activity over respective controls was observed for all e-liquids and extracts (Figure 1B). The level of e-liquids as high as 15-fold higher than SLTs and NRT extracts did not induce any activity. No evidence of cytotoxicity, as determined by the background bacterial lawn, was observed for all e-liquid, SLT and NRT samples at all doses tested.

### 3.3. E-Liquids, Smokeless Tobaccos *(*SLTs*)* and Nicotine Replacement Therapy *(*NRT*)*: Genotoxicity

The results of *in vitro* MN formation assay are shown in Figure 1C,D). The MN assay conducted in CHO-K1 cells, identifies clastogenic and aneugenic chemicals which essentially cause a DNA-damaging event that leads to the disruption or breakage of chromosomes and ultimately results in sections of the chromosome being deleted, added, or rearranged upon cell division (mitosis) [29].

A sample dose range which does not produce cytotoxicity of more than 55 ± 5% (compared to control) was utilized in the MN formation assay [24]. The control e-cig, e-liquids, SLT and NRT extracts did not induce any significant cytotoxicity at all dose levels tested since cell-viability remained around 100% of control at all concentrations (Figure 1D). No significant induction in the MN formation over respective controls was observed for all e-liquids and SLT and NRT extracts (Figure 1C).

### 3.4. E-Liquids, Smokeless Tobaccos *(*SLTs*)* and Nicotine Replacement Therapy *(*NRT*)*: Inflammation

Airway epithelial cells are the first line of defense in the airways to respond to any external stimuli and secrete specific chemo-attractants and pro-inflammatory cytokines, for example IL-8, monocyte chemotactic protein-1, and IL-1ß, in order to activate the secondary response for neutrophils and macrophage infiltration [30,31]. Instead of measuring downstream acute or chronic phase inflammation specific cytokines, this study measured an upstream pro-inflammatory cytokine, IL-8.

**Figure 3:**
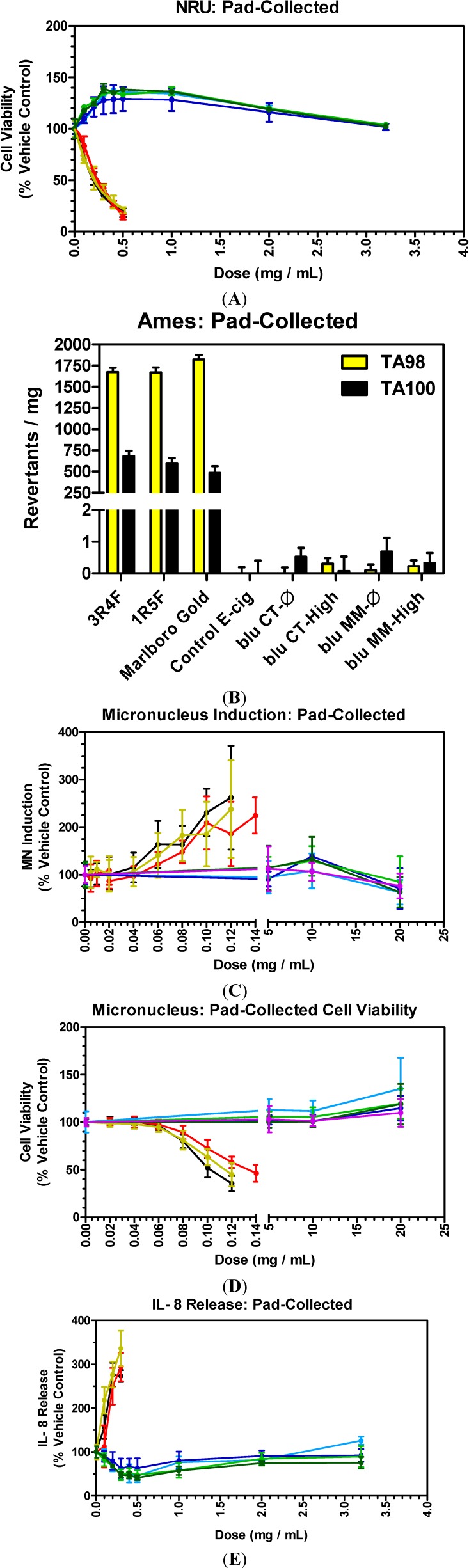
*In vitro* activity of pad-collected WTPM from tobacco cigarettes and pad-collected e-cig aerosols in NRU (**A**), Ames (**B**), MN (**C** and **D**), and IL-8 (**E**). NRU, MN and IL-8 data is reported as % vehicle control; PBS in the case of e-cigarette pad-collected aerosols, DMSO for tobacco-burning cigarette pad-collected WTPM. Control e-cig exposures in NRU and IL-8 were at the highest deliverable dose, resulting in no observable cytotoxicity or IL-8 release above background levels (data not shown). Data points in each plot represent the mean values ± SD from a minimum of two (2) independent experiments. MN cell viability (**D**) shown to verify lack of MN induction is not due to cytotoxicity at the higher doses. (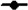) 3R4F; (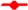) 1R5F; (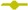) Marlboro Gold; (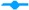) blu CT-Ø; (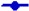) blu CT-High; (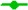) blu MM-Ø; (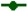) blu MM-High; (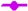) Control e-cig.

The effects of 24 h exposures of e-liquids and SLT and NRT extracts on IL-8 release in A549 cells are shown in Figure 1E. The control samples for e-cig, SLTs and NRT did not induce any significant IL-8 release. No significant IL-8 release was observed for most of the products, with the exception of the blu MM-Ø, blu MM-High and blu CT-Ø treatments which resulted in higher IL-8 release only at extremely high doses of 6.9–13.8 mg/mL. When compared to the IL-8 release induced by conventional cigarette samples (Figure 3E), any significant IL-8 release as a result of the blu MM e-liquid treatments occurred at doses approximately 42-fold higher than the conventional tobacco cigarettes. It has been suggested that the toxicity of e-liquids may change when the same e-liquids are heated to produce the inhaled aerosol [26]. The evaluation of e-cig aerosol toxicity is essential since the intended use of e-cigarettes is through aerosol inhalation. Additionally, it is proposed that different e-liquid formulation ingredients may evaporate differently, leading to changes in concentrations in the generated aerosols as well as the possibility that components may undergo modification when subjected to the heat used to generate the aerosol; therefore, the final composition of the aerosol may be different when compared to the e-liquid [9]. In the light of that, the purpose of the study was to also characterize the aerosol toxicity as delivered by heating the e-liquid.

### 3.5. E-Cigarettes and Conventional Cigarettes

In order to study the comparative toxicities of the e-cig aerosols and tobacco smoke, all products were smoked by the standardized CI profile with the aerosols or smoke from each product being collected on a pad as described in the Experimental Section. The pad-collected tobacco smoke matter was extracted in DMSO because it has been widely applied as a vehicle for *in vitro* assays of test articles of limited water solubility due to its excellent solvent properties for both polar and non-polar compounds and its moderate toxicity to test organisms [32,33]. The reasoning for the concentration ranges utilized in this study was to limit the level of DMSO in order to avoid any solvent specific effects on the assays [32]. The toxicological responses of the pad-collected e-cig aerosols and cigarette smoke are shown in Figure 3A–E.

### 3.6. E-Cigarettes and Conventional Cigarettes: Cytotoxicity

The cytotoxicity of e-cig pad-collected aerosol is shown in Figure 3A. The e-cig pad-collected aerosol was not cytotoxic at all tested levels. For comparative purposes, different levels of WTPM from tobacco cigarettes were also tested. A dose-dependent increase in cell death was observed for 3R4F, 1R5F and Marlboro Gold cigarettes with up to 90% cell death at the 0.5 mg/mL maximum applied dose. The WTPM mediated cytotoxicity results are in agreement with previously reported studies [12,34]. It was not possible to quantify the comparative cytotoxicity in terms of traditional EC_50_ values (a dose which induces 50% cell death) since no cell death was observed at any concentration used for all e-cig samples (Table 2 and Figure 3A).

There was an observed increase in cellular viability in cells treated with e-liquids (Figure 1A) and lower doses of pure nicotine (Figure 2A). Also treatment with e-cig aerosols resulted in a similar increase in cellular viability (Figure 3A). This observed increase in cellular viability for pure nicotine and both e-cig liquids and pad-collected aerosols could be related to nicotine’s effect on cellular proliferation and protection [27].

**Table 2:** NRU EC_50_ values for WTPM only (mean ± SE). EC_50_ expressed in mg/mL to correct for differences in dose volumes between exposure methods. ^†^ ND: e-cig pad-collected aerosols EC_50_ not determined since cytotoxicity was not detected at doses tested.

Pad-Collected Matter: Smoke and Aerosols		
Sample	NRU EC_50_ (mg/mL)	S.E.
3R4F	0.196	0.010
1R5F	0.237	0.014
Marlboro Gold	0.204	0.009
Control e-cig	ND ^†^	--
blu CT-Ø	ND ^†^	--
blu CT-High	ND ^†^	--
blu MM- Ø	ND ^†^	--
blu MM-High	ND ^†^	--

Similar findings were reported for aqueous extracts of aerosols from various commercial e-cigs studied in cultured mammalian fibroblast and myocardial cells [9,10]. Both studies also reported that some aqueous extracts of e-cig aerosols showed cytotoxicity related to flavors, but were significantly less cytotoxic than cigarette smoke extracts. This study evaluated two flavored e-cigs, with and without nicotine (Table 1).

With e-liquids and pad-collected aerosols, no nicotine or flavor specific cytotoxic effects were evident with e-cigs without nicotine, blu MM-Ø, and blu CT-Ø, and with nicotine as high as 24 mg/mL, blu MM-High and blu CT-High (Figure 1A and Figure 3A). The cytotoxicity and inflammatory response of L-nicotine in this cell culture system (Figure 2) was also tested. No cytotoxicity and inflammation (IL-8 release) were observed below 2.5 mg/mL and 1.0 mg/mL nicotine, respectively (Figure 2A,B). The highest level of nicotine in e-liquids tested was about 0.5 mg/mL (Table 1), which was below the concentrations of L-nicotine required to induce cytotoxicity and IL-8 release in this experimental method. In addition, the WTPM from tobacco cigarettes at 0.5 mg/mL was extremely cytotoxic (Figure 3A), corresponding to a nicotine concentration of approximately 0.04 mg/mL, which was well below nicotine mediated toxic levels, demonstrating that WTPM induced cytotoxicity was not mediated by nicotine.

### 3.7. E-Cigarettes and Conventional Cigarettes: Mutagenicity

The mutagenicity of pad-collected smoke and aerosols of tobacco cigarettes and e-cigs, respectively, is shown in Figure 3B. No activity was observed for control e-cig samples in the Ames assay. The specific activity for all tobacco cigarettes was in the range of 1600–1850 and 500–750 revertants/mg WTPM for TA98 and TA100, respectively (Figure 3B). Historically, WTPM prepared under the same smoking conditions has been shown to have similar levels of specific activity (revertants/mg) [35].

No increase in Ames activity was observed for any e-cigs used in this study and the revertants/mg was extremely low and within assay background measurements (< 2 revertants/mg). It was not possible to quantify the specific activities for e-cig aerosols since no increase in revertant counts was observed with increasing doses for all tested e-cig samples (Figure 3B).

### 3.8. E-Cigarettes and Conventional Cigarettes: Genotoxicity

The cell viability and clastogenic effects of WTPM and e-cig pad-collected aerosols are presented in Figure 3C,D. The pad-collected aerosol from the control e-cig containing glycerol/water and the solvent (DMSO) control did not induce any MN formation. A significant dose-dependent WTPM mediated induction in MN formation was observed with all tobacco cigarettes (3R4F, 1R5F and Marlboro Gold). No increase in the MN formation was observed for pad-collected aerosols from e-cigs at all doses tested (Figure 3C). The maximum induction in the MN formation with tobacco cigarettes was about 2.5 to 3-fold over background at about 0.12 mg/mL WTPM. A sharp decrease in the WTPM-induced MN formation was observed at dose levels higher than 0.12 mg/mL WTPM due to the decrease in cell viability (Figure 3D). Similar MN findings have been reported [31]. The dose at which tobacco cigarette WTPM induced maximum MN formation is approximately 166-fold lower than the maximum e-cig pad-collected aerosol dose (20 mg/mL) tested, which had no observed induced MN formation.

### 3.9. E-Cigarettes and Conventional Cigarettes: Inflammation

The inflammatory responses, as measured by IL-8 release from cells treated with tobacco WTPM and e-cig aerosols are presented in Figure 3E. No IL-8 release was observed for control samples. The pad-collected aerosols from all e-cigs did not induce any IL-8 release at all doses tested (Figure 3E). In contrast, a WTPM mediated dose-dependent increase in IL-8 release was observed for all tobacco cigarettes. A sharp decrease in the IL-8 level at WTPM levels over 0.3 mg/mL was noticed (data not shown) since significant cytotoxicity was observed at those doses (Figure 3A).

The WTPM dose at which a significant IL-8 release was observed (0.15 mg/mL) was about 20-fold lower than the maximum e-cig pad-collected aerosol tested dose (3.2 mg/mL), at which no inflammatory effect was observed. The release of IL-8 in cultured cells by different cigarette smoke preparations has been reported [36] and this inflammatory response has been associated with oxidative stress due to the free radicals present in cigarette smoke [37].

At the lower doses of e-cig pad-collected aerosols utilized in this study, compared to the control, a lower release of IL-8 was observed (Figure 3E). That effect was also evident in cells treated with lower doses of pure nicotine (Figure 2B). This phenomenon of a lower release of an inflammatory cytokine (IL-8) is associated with the anti-inflammatory effects of nicotine [38]. There may be an association between the lower nicotine present in e-cig pad-collected aerosols and anti-inflammatory effects.

### 3.10. Nicotine Equivalence

Assessment of *in vitro* responses observed in this study was also calculated based on the level of nicotine present in the test samples. The level of nicotine concentrations measured in the prepared samples are shown in Table 1 but varied depending on sample volume and toxicity endpoint measured in this study. The upper limit of nicotine measured in tobacco cigarette WTPM was about 0.025 mg/mL, e-cig pad-collected aerosol was about 0.223 mg/mL and e-liquid was about 0.522 mg/mL. Thus, the nicotine concentration was about 10 to 20-fold higher in e-cig samples as compared to conventional cigarette samples. No nicotine mediated cellular toxicity was observed at 2.5 mg nicotine/mL (Figure 2A). At low doses up to 2.0 mg/mL, L-nicotine in fact increased the cellular proliferation as indicated by the higher cell viability than the control (Figure 2A) indicating a cellular protective response [27] as well as lower release of IL-8 in the media than control (Figure 2B) suggesting the anti-inflammatory properties of nicotine [38].

The average human exposure to nicotine on a 10-puff basis from a typical tobacco cigarette (tar 11.4 ± 0.1 mg/cig) is about 2.0 mg and from an e-cig product (labeled 24 mg nicotine) is about 0.23 mg under the similar CI smoking profile used for this study [39]. Therefore, it was evident that the nicotine levels in e-cig treatments were well below the toxic level of L-nicotine and represent comparative nicotine levels present in the tested e-cig and conventional cigarette samples. In addition, based on literature on the beneficial role of nicotine, relative to e-liquids and pad-collected aerosols used in this study and pure nicotine effects, there may be an association between lower levels of nicotine present in samples used in this study and cellular protection as well as anti-inflammatory effects [27,38].

Under the experimental conditions used to evaluate traditional tobacco burning cigarettes, e-cigs did not produce any meaningful toxic effects as measured by four *in vitro* endpoints. These results demonstrate the potential for e-cigs to significantly reduce the toxicological impact when compared to traditional tobacco burning cigarettes.

### 3.11. Comparable Human Exposure: Conventional and E-Cig

The comparative human exposure to tobacco cigarette smoke and e-cig aerosol is important in order to assess e-cig mediated reduced exposure and reduced harm. It has been reported that a smoker with one pack-a-day tobacco cigarette consumption inhales on average about 261 mg/m^3^ cigarette tar [40], equivalent to about 271 µg/mL or 0.271 mg/mL [40]. Internal study indicated the range of e-cig aerosol delivery to be in the range of 0.5–1.5 mg/puff under Canadian Intense conditions (39). Assuming similar e-cig use as a conventional tobacco cigarette (200 puffs), the upper limit of human exposure to e-cig aerosol is approximately 300 mg or approximately 250 µg/mL or 0.25 mg/mL. The range of e-cig pad-collected aerosol used in the present study was 3.2–20 mg/mL. No adverse toxicological events were observed in this study even when the e-cig aerosol levels used were about 12–78 times higher than expected with normal e-cig use.

### 3.12. Contribution of Findings to Tobacco Harm Reduction and E-Cigs

The concept of Tobacco Harm Reduction (THR) has been advanced as a pragmatic approach to achieving reductions in the adverse public health impacts of cigarette smoking in the near term; in parallel with social, educational, and regulatory strategies intended to reduce and discourage cigarette smoking, particularly among adolescents [4,41,42,43].

The use of non-combustible SLT products such as Swedish-style snus and traditional moist snuff are demonstrably on the order of 98% less harmful in terms of risks for lung cancer, COPD, CVD, and other cancers, (including oral cancers) as compared to cigarette smoking [11,41,44,45].

Similarly, NRT products such as dermal patches and chewing gums have been shown to be safe and efficacious in clinically-managed and over-the-counter consumer usage. Therapeutic nicotine vapor inhaler devices and aerosol sprays for nasal and oral use have to date demonstrated similar benefits and low risks in facilitating smoking cessation [46]. The efficacy of such conventional NRT cessation aids has, however, proven in practice to fall short (~7% cessation success) of what is needed by a considerable number of smokers [47]. These smokers consistently report that taste, sensory and behavioral components experienced in the act of cigarette smoking are substantial motivators of the smoking behavior and may well comprise a population that could achieve substantially higher success in quitting through use of products that mimic the behavior element of smoking as well as the delivery of nicotine. Therefore, the use of e-cigs that provide some of the taste, sensory and behavioral components of conventional tobacco cigarette smoking may hold substantial promise in defining the potential benefits of the THR paradigm [48].

Despite the absence of long-term epidemiologic data on any chronic disease risk, a growing body of recent literature is consistent with an expectation that the use of e-cigs is unlikely to raise serious health concerns [49,50], particularly in comparison to those that result from the smoking of conventional cigarettes [7]. This conclusion is currently based, and further supported by this study and on a growing number of independent analyses of commercially-available e-cig liquids and product aerosols from markets around the world, that have consistently reported very low or undetectable levels of most tobacco smoke constituents that are known or suspected to play a prominent role in the etiology of serious tobacco-related diseases. [7,51,52,53,54,55,56,57,58,59].

There are various essential and contributory components to the THR framework, including product use and behavior, taste, nicotine delivery, product chemistry, toxicity and clinical safety. This study shows that neither the e-cig liquids nor collected aerosols produced any meaningful toxic effects in widely used *in vitro* test systems. These findings add additional value to the increasing body of scientific weight-of-evidence supporting the potential inclusion of e-cigs into THR paradigm.

## 4. Conclusions

In summary, this comparative *in vitro* toxicity study of e-cigs, SLT, NRT and tobacco cigarette products demonstrates the following:

(1) E-cigs *vs*. Tobacco WTPM: At doses up to approximately 100-fold higher than typical cigarette smoke exposures, blu e-cig liquids and pad-collected aerosols had no-to-extremely low *in vitro* activity (NRU, Ames, MN and IL-8) when compared to WTPM from tobacco burning cigarettes. WTPM activity was up to approximately 6,000 times higher than e-cigs.
(2) E-cigs *vs*. SLT and NRT: blu e-cig liquids demonstrated similar no-to-extremely low *in vitro* activity as aqueous extracts from a commercial nicotine lozenge (NRT) and commercial SLT products (snus and snuff).
(3) Effect of Nicotine: *In vitro* activities (NRU, Ames, MN and IL-8) measured for blu e-cig exposures, with and without nicotine, were similar for all sample types, indicating that the presence of nicotine, at the levels tested, did not contribute to any toxicological effects, confirmed by the lack of cytotoxicity and inflammation response of L-nicotine at comparative levels.
(4) Effect of Flavors: *In vitro* activities (NRU, Ames and MN) for the commercial blu e-cigs were indistinguishable from control (glycerol/water); indicating these flavors (CT and MM), at the levels tested, had no detectable impact on the cytotoxicity and genotoxicity endpoints utilized in this study. There was some observed IL-8 induction for some e-liquids, albeit at the highest doses tested.
(5) Liquid *vs*. Pad-Collected Aerosol: *In vitro* results for blu e-cigs, in this study, were similar for the different exposure methods (e-liquids and pad-collected aerosol); demonstrating no detectable impact on the *in vitro* toxicological responses when the e-liquids were aerosolized.
(6) SLT *vs*. Tobacco WTPM: SLT extracts added to the test systems at levels up to 54-fold higher than those used for Tobacco-WTPM generated by burning cigarettes was markedly less cytotoxic and mutagenic, and evoked a significantly lower IL-8 response at all dose levels evaluated. The effects of the SLT extracts in the assays were statistically indistinguishable from those of the e-cig and NRT preparations.

With respect to the study, lack of any meaningful *in vitro* acute toxicity for blu e-cigs and extremely low levels of chemical constituents measured in blu [39] and the analysis of known reduced risk products such as NRT and SLT has the potential to demonstrate a decreased human health impact as compared to conventional tobacco-burning cigarettes.
